# The Role of Photobionts as Drivers of Diversification in an Island Radiation of Lichen-Forming Fungi

**DOI:** 10.3389/fmicb.2021.784182

**Published:** 2022-01-03

**Authors:** Miguel Blázquez, Lucía S. Hernández-Moreno, Francisco Gasulla, Israel Pérez-Vargas, Sergio Pérez-Ortega

**Affiliations:** ^1^Department of Mycology, Real Jardín Botánico (CSIC), Madrid, Spain; ^2^Open Access Publication Support Program, Consejo Superior de Investigaciones Científicas (CSIC), Madrid, Spain; ^3^Escuela Internacional de Doctorado, Universidad Rey Juan Carlos, Móstoles, Spain; ^4^Department of Life Sciences, Universidad de Alcalá, Alcalá de Henares, Spain; ^5^Department of Botany, Ecology and Plant Physiology, Universidad de La Laguna, San Cristóbal de La Laguna, Spain

**Keywords:** Macaronesia, *Ramalina*, trophic niche, metabarcoding, adaptive radiation

## Abstract

Speciation in oceanic islands has attracted the interest of scientists since the 19th century. One of the most striking evolutionary phenomena that can be studied in islands is adaptive radiation, that is, when a lineage gives rise to different species by means of ecological speciation. Some of the best-known examples of adaptive radiation are charismatic organisms like the Darwin finches of the Galapagos and the cichlid fishes of the great African lakes. In these and many other examples, a segregation of the trophic niche has been shown to be an important diversification driver. Radiations are known in other groups of organisms, such as lichen-forming fungi. However, very few studies have investigated their adaptive nature, and none have focused on the trophic niche. In this study, we explore the role of the trophic niche in a putative radiation of endemic species from the Macaronesian Region, the *Ramalina decipiens* group. The photobiont diversity was studied by Illumina MiSeq sequencing of the ITS2 region of 197 specimens spanning the phylogenetic breadth and geographic range of the group. A total of 66 amplicon sequence variants belonging to the four main clades of the algal genus *Trebouxia* were found. Approximately half of the examined thalli showed algal coexistence, but in most of them, a single main photobiont amounted to more than 90% of the reads. However, there were no significant differences in photobiont identity and in the abundance of ITS2 reads across the species of the group. We conclude that a segregation of the trophic niche has not occurred in the *R. decipiens* radiation.

## Introduction

Oceanic islands, which are of volcanic origin and have never been connected to continental land masses, have attracted the interest of scientists since the 19th century, using them as natural laboratories in which to study the origin of biodiversity and its evolution (Whittaker and Fernández-Palacios, 2007; Warren et al., 2015). Perhaps the best-studied evolutionary phenomenon on oceanic islands has been that of cladogenetic speciation, that is, how from a colonization event a lineage radiates into a collection of species (Gould and Eldredge, 1977).

Radiations can be adaptive or non-adaptive (Gittenberger, 1991; Schluter, 2000; Rundell and Price, 2009). Non-adaptive radiations are those in which diversification is not accompanied by niche differentiation and usually gives rise to ecologically similar allopatric species (Gittenberger, 1991; Rundell and Price, 2009). On the other hand, in adaptive radiations, ecological speciation is the driver of diversification, and niche differentiation among radiation-originating species allows sympatric patterns (Schluter, 2000). One key point in defining an adaptive radiation is that the traits presented by the species (phenotype) must have a clear correlation with the environment in which the species occur (Schluter, 2000; Givnish, 2015). Therefore, in the study of the adaptive nature of a radiation, it is essential to study the variability of functional traits in the species of the radiation and their correlation with abiotic factors. The best-studied examples of adaptive radiation have focused on charismatic organisms, such as the Darwin finches of the Galápagos Islands (Grant and Grant, 2008), the silversword alliance of Hawaii (Landis et al., 2018), or the *Anolis* lizards of the Greater Antilles (Losos, 2009). However, the number of studies that have investigated the adaptive nature on radiations of other groups of less charismatic organisms is still very limited (Gaya et al., 2015; Mao et al., 2018).

Lichens are the symbiotic phenotype of fungi with a specialized type of nutrition that takes sugars from a population of extracellular algae and/or cyanobacteria (hereafter, the photobionts). Thus, for lichen-forming fungi (hereafter, the mycobionts), the identity and abundance of their associated photobionts constitute the trophic dimension of their ecological niche. Although more than 50 genera of algae and cyanobacteria are known to act as lichen photobionts (Friedl and Büdel, 2008; Sanders and Masumoto, 2021), the vast majority of the nearly 20,000 described species of lichen-forming fungi (Lücking et al., 2017) are associated with algae from the class *Trebouxiophyceae* (Friedl and Büdel, 2008). Among the *Trebouxiophyceae*, the species of the genus *Trebouxia* are the most common photobionts (Friedl and Büdel, 2008; Muggia et al., 2020). The role of photobionts as drivers of speciation in lichen-forming fungi is still poorly known. There are several factors that may determine the contribution of photobionts in mycobiont diversification (Singh et al., 2017). On the one hand, it has been proposed that photobiont niches are influenced by abiotic conditions and these preferences may limit lichen distributions (Peksa and Škaloud, 2011). It has been shown that photobiont switches between physiologically compatible photobionts are mechanisms to widen the mycobiont ecological niches and geographic ranges (Fernández-Mendoza et al., 2011; del Campo et al., 2013; Rolshausen et al., 2018, 2020) or for ecological speciation (Ortiz-Álvarez et al., 2015). On the other hand, not all mycobiont species show similar specialization toward their photobionts (Pérez-Ortega et al., 2012; Magain et al., 2017), ranging from narrow specialists (Garrido-Benavent et al., 2017) to generalists (Muggia et al., 2014). In addition, different *Trebouxia* species have been found to coexist inside a single lichen thallus (Piercey-Normore, 2006; del Campo et al., 2010; Muggia et al., 2013). These species show differences in various aspects of their physiology, such as growth rate (Casano et al., 2011), photosynthetic output (Casano et al., 2011), response to oxidative stress (del Hoyo et al., 2011), and heavy metal tolerance (Álvarez et al., 2015). It is still not clear whether coexistence is a widespread phenomenon or it is reduced to certain species or lineages, and its significance in ecological and evolutionary times.

*Ramalina* is a well-known genus of lichen-forming fungi which forms usually large fruticose thalli of a more or less light-yellow color. The genus belongs to a clade of fruticose genera in the Ramalinaceae (Spjut et al., 2020), a family dominated by microlichens (Kistenich et al., 2018). The genus is subcosmopolitan and with c. 230 species is one of the largest genera of lichen-forming fungi (Lücking et al., 2017). Most of its diversity is concentrated in five areas: The Andes (Marcano et al., 2021), East Africa (Krog and Swinscow, 1976), Baja California (Bowler and Rundel, 1972, 1973), Australasia (Stevens, 1987; Blanchon et al., 1996) and Macaronesia (Krog and Østhagen, 1980a,b; Krog, 1990). Macaronesia is a group of volcanic archipelagos in the North Atlantic: Azores, Cape Verde, Canary Islands, Madeira, and Selvagens Islands. In spite of the small area, a total of 37 species of *Ramalina* have been recorded from the area, of which 25 are endemic (68%, most outstanding being the island of Porto Santo which, with an area of 42 km^2^, hosts a total of 6 endemic *Ramalina* species (Sparrius et al., 2017). Among the endemic species of *Ramalina* in Macaronesia two groups of saxicolous species stand out, the *R. bourgaeana* group and the *R. decipiens* group, which are defined by the presence of characteristic structures, chondroid strands embedded in the medulla in the former and adjacent to the cortex in the later (Krog and Østhagen, 1980a; Spjut et al., 2020). The taxonomy of the *Ramalina decipiens* group has been recently revisited through an integrative approach recognizing 15 species-level lineages, six of which are newly described and two remain formally undescribed (Blázquez et al., in prep.). Of the fifteen recognized species, all are endemic to Macaronesia, except *R. maderensis*, which has also been reported from Saint Helena (Aptroot, 2008). In addition, most of them are single-island endemics, but two species (*R. decipiens* and *R. maderensis*) are widespread.

In this study, we hypothesize that photobiont-mycobiont associations play an important role in adaptive radiations of lichen-forming fungi. Photobiont differences among the species of a radiating clade of mycobionts, either in identity or abundance, could indicate a segregation of the trophic niche of the clade. We propose the *Ramalina decipiens* group as a framework to study the role of trophic niche segregation as a key driver in adaptive radiation. In order to explore and test this hypothesis, we are to analyze (i) the diversity of the photobionts associated with the species of the *R. decipiens* group, (ii) the relative importance of mycobiont identity, island, and macroclimate on photobiont community structure, (iii) the level of specialization of each *Ramalina* species toward its photobionts and (iv) whether specialization and photobiont diversity are phylogenetically structured. In addition, as algal coexistence has been previously reported in *Ramalina* species, we also intend (v) to explore to what extent this phenomenon occurs in the group.

## Materials and Methods

### Sampling

We studied 197 specimens of the *R. decipiens* group representing the 15 species-level lineages (Blázquez et al. in preparation). Seven of those species correspond to previously known species (Krog and Østhagen, 1980a,b; Krog, 1990). Another six represent new species which are going to be described in Blázquez et al. (in preparation). They are referred here with the following provisional names: *R. delicata* nom. Prov., *R. fortunata* nom. Prov., *R. gomerana* nom. Prov., *R. papyracea* nom. Prov., *R. sabinosae* nom. Prov., and *R. sampaiana* nom. Prov. Two further species-level lineages will remain unnamed until further material is available and are referred in the present study as *Ramalina* sp. 1 and *Ramalina* sp. 2. The specimens were collected throughout 66 localities spanning the seven Canary Islands, the islands of Madeira and Porto Santo of the Madeira archipelago, and the islands of Sal and São Vicente of the Cape Verde archipelago (Supplementary Table 1).

### DNA Extraction, Amplification, and Sequencing

A small thallus fragment from each specimen (c. 10 mm^2^) was excised from the tip of a single laciniae with the help of razor blade and forceps under a Nikon SMZ800 stereomicroscope. First, we inspected the thalli under a x80 magnification to check for areas free of epiphytic microalgae and fungi. Thallus tips were the only zones free of epiphytes in all samples. The choice of thallus tips is also supported by the finding of Molins et al. (2021), who reported that there were no differences in photobiont composition among different parts of the thallus in specimens of a species of the same genus (*R. farinacea*) from the same biogeographic region as the present study. Samples were washed with acetone to remove secondary metabolites and stored at −80°C. After 1 h of freezing, they were pulverized using TissueLyser II (Qiagen) with two crystal beads. Genomic DNA was extracted using E.Z.N.A.® Forensic DNA Kit (Omega Bio-Tek), following the instructions of the manufacturer. The second region of the ribosomal internal transcriber spacer (ITS2) was used as barcode to prospect photobiont diversity. We used primers FDGITS2-f and FDGITS2-r (Dal Grande et al., 2018) for amplification. These primers included Fluidigm CS1 and CS2 universal oligomer sequences at their 5′ ends. PCR reactions were carried out in a total volume of 15 μl, containing 3 μl of template DNA, 0.3 μl of each primer (10 μM), 0.6 μl of MyFi DNA Polymerase (Bioline, Sydney, Australia), 3 μl of 5x MyFi Reaction Buffer, and 7.8 μl of distilled water. PCR settings consisted in an initial denaturation at 95°C for 1 min; 35 cycles of 95°C for 15 s, 54°C for 15 s, and 72°C for 15 s; with a final extension at 72°C for 5 min. PCR products were checked in 1% agarose gels stained with SYBR™ Safe DNA Gel Stain (Thermo Fisher Scientific). PCR products were quantified using the Qubit dsDNA HS (High Sensitivity) Assay Kit (Thermo Fisher Scientific) and pooled in equimolar concentrations for sequencing on a single MiSeq run (Illumina, United States) at the RTSF Genomics Core at Michigan State University (East Lansing, Michigan). The Genomics Core completed library preparation by secondary PCR targeting the Fluidigm CS1/CS2 oligos at the ends of the primary PCR products. Primers used for the second PCR included dual indexed, Illumina library compatible sequences. Finished libraries were bulk normalized using Invitrogen SequalPrep DNA Normalization Plates (Thermo Fisher Scientific) and the recovered products pooled. The pool was QC’d and quantified using a combination of Qubit dsDNA HS (Thermo Fisher Scientific), Agilent 4,200 TapeStation HS DNA1000 (Agilent Technologies), and Kapa Illumina Library Quantification qPCR assays (Kapa Biosystems, Inc.). This pool was loaded onto an Illumina MiSeq v2 Standard flow cell and sequencing performed in a 2x250bp paired-end format using a MiSeq v2 500 cycle reagent cartridge. Custom sequencing and index primers complementary to the Fluidigm CS1/CS2 oligos were added to appropriate wells of the reagent cartridge. Base calling was done by Illumina Real-Time Analysis (RTA) v1.18.54, and output of RTA was demultiplexed and converted to FastQ format with Illumina Bcl2fastq v2.19.1.

### Sequence Processing

Raw sequence data were processed using the DADA2 pipeline (Callahan et al., 2016) in R 4.0.3 (R Core Team, 2020). DADA2 takes a set of demultiplexed paired-end fastq files, filters the sequences based on their length and quality, assembles them into error-corrected amplicon sequence variants (ASVs), and removes chimeric ASVs. We chose this pipeline because ASVs make possible to work at a finer resolution than other approaches based on the creation of operational taxonomic units (OTUs). Besides, ASVs allow for greater reproducibility, reusability, and comprehensiveness than OTUs (Callahan et al., 2017). Following Dal Grande et al. (2018) we considered that ASVs that represented <0.005% (i.e., n = 100) of the total reads were most probably exogenous to the symbiosis and did not include them in downstream analyses. We then assigned taxonomy to the ASVs using all sequences assembled by Muggia et al. (2020) as a taxonomic reference. This was done using the *assignTaxonomy* function in DADA2. Given that microalgae can show ecophysiological differences even when they are phylogenetically close (Sadowsky et al., 2016) we chose to conduct all analyses in this study at the ASV level. All sequences obtained in this study are available in the SRA (NCBI) under BioProject PRJNA764073.

### Alignment, Phylogenetic Relationships, and ASV Haplotype Networks

ASVs were aligned using MAFFT v.7.308 (Katoh et al., 2002) as implemented in Geneious® v.9.1.8. Sequences of *Asterochloris glomerata* (MW043487, Pino-Bodas and Stenroos, 2021) and *Vulcanochloris guanchorum* (KR952330, Vančurová et al., 2015) were included as outgroup. We inferred phylogenetic relationships among ASVs using Maximum Likelihood using RAxMLHPC2 v. 8.2.4 (Stamatakis, 2014) as implemented in CIPRES Science Gateway (Miller et al., 2011). We selected the GTRGAMMA substitution model and carried out 1,000 rapid bootstrap pseudoreplicates to evaluate nodal support. We considered nodes with bootstrap values equal or higher than 70% to be significantly supported. The resulting phylogenetic tree was visualized in FigTree 1.4.4^1^ and edited using Adobe Illustrator CS5 (Adobe Systems Inc., San José, United States). Additionally, we generated haplotype networks for the *Trebouxia* species represented by more than three ASVs. We used the TCS method (Templeton et al., 1992) as implemented in PopART v.1.7 (Leigh and Bryant, 2015) for network construction. These networks were edited in Adobe Illustrator CS5 to show the prevalence of the different recovered ASV in each sampled *Ramalina* species and island.

### Differences in Frequency of ASVs in Islands and Species

To explore whether the frequency of ASVs was different among islands and *Ramalina* species we adjusted a two-way ANOVA for each ASV. ASV frequency was used as the response variable and island and *Ramalina* species as explanatory variables. We used Levene’s test to check whether data for each ASV met the normality assumption necessary to carry out a two-way ANOVA. In the cases in which our data did not meet the assumptions, we employed the non-parametric Kruskal-Wallis test. This was done using the functions *aov*, *leveneTest*, and *kruskal.test* in base R 4.0.3 (R Core Team, 2020). ASVs occurring only in one specimen were not included in this analysis.

### Photobiont Community Structure

In order to explore the relative importance of mycobiont identity, macroclimate, and island in shaping photobiont communities we performed distance-based redundancy analysis (dbRDA, Legendre and Anderson, 1999). Following Shankar et al. (2017), we calculated an abundance-weighed phylogenetic UniFrac distance-based dissimilarity matrix. This was done with the R package *phyloseq* (McMurdie and Holmes, 2013). We transformed the ASV table into relative ASV abundances using the function *decostand* of the *vegan* package (Oksanen et al., 2012) with the method “total” and combined it with the taxonomy of the ASVs and the RAxML phylogeny previously generated into a phyloseq object using the *merge_phyloseq* function. The dissimilarity matrix was calculated from this object using the *UniFrac* function. The weighed UniFrac dissimilarity matrix was used as the response matrix in the dbRDA. We used three explanatory matrices: (1) first matrix corresponded to the *Ramalina* species, (2) second to the island where samples were collected, (3) and third matrix included five macroclimatic variables downloaded from WorldClim (Fick and Hijmans, 2017): average temperature (°C), precipitation (mm), solar radiation (kJ m^−2^ day^−1^), wind speed (m s^−1^), and water vapor pressure (kPa). Because lichens are photosynthetic and poikilohydric symbioses, we chose to use these instead of the 19 widely used bioclimatic variables also available in WorldClim, that are derived from temperature and rainfall only. All layers of the five variables (data for each month of the year are available) were downloaded at a resolution of 30 s (~1 km^2^) and merged into year averages using the *calc* function of the *raster* R package (Hijmans et al., 2015). Then, variable values for each sample were obtained through their geographic coordinates using the *extract* function from the *raster* package. Before computing the dbRDA we checked for correlation between WorldClim variables in our samples. We calculated a Pearson correlation coefficient matrix and generated a correlation hierarchical cluster plot with the absolute correlation values. Precipitation and water vapor pressure were dropped as they were correlated (absolute correlation >0.5) with solar radiation and average temperature, respectively. Then we inspected the remaining variables to check their normality. To do so we generated a histogram for each variable with the *hist* function of base R. Solar radiation was found to be not normal and was log-transformed. dbRDA was then calculated with the *dbrda* function of the *vegan* R package (Oksanen et al., 2012). Variance partitioning between the three explanatory matrices was estimated using adjusted R^2^. Adjusted R^2^ significance was assessed for each fraction with a permutation-based ANOVA test with 2000 permutations. This was done in *vegan* using the functions *varpart* and *anova.cca*. Significance of the dbRDA as a whole was also assessed with a permutation-based ANOVA test with 2000 permutations, this time with the function *anova*. To visualize variance partitioning we generated a Venn diagram plotting the output of the *varpart* function.

### Algal Co-occurrence Analyses

Based on the ASVs abundance table we calculated for each *Ramalina* species: number of studied thalli, percentage of thalli that showed algal coexistence, minimum number of ASVs in one thallus, mean number of ASVs per thallus, and maximum number of ASVs in one thallus. For the species that showed algal coexistence, we also calculated: minimum relative abundance of the main photobiont found in one thallus, mean relative abundance of the main photobiont across all thalli showing algal coexistence and maximum relative abundance of the main photobiont found in one thallus.

### Bipartite Network, Specialization, and Modularity

We built two different adjacent matrices *i × j*, where *i* represents mycobionts and *j* ASVs. In the first matrix, interaction frequency between mycobiont *i* and ASV *j* was calculated as the sum of reads for ASV *j* found in all specimens of mycobiont *i* previously rarefied to the depth of the sample with the lower number of reads (Weiss et al., 2017). In the second matrix, number of read are not taken into account and the interaction frequency was based in the number of specimens of the mycobiont *i* where ASV *j* was found. The rarefaction was done with the *rarefy_even_depth* function of the *phyloseq* (McMurdie and Holmes, 2013) R package. The presence-absence conversion was done using the function *decostand* of the *vegan* package (Oksanen et al., 2012) with the method “pa.” For each network, we calculated the parameter *d’* (Blüthgen et al., 2006) to estimate the specialization of the mycobionts toward the ASVs. This index ranges from 0 (no specialization) to 1 (high specialization). We also calculated species degree, which is the sum of links per species, and *ND*, which is species degree normalized by the number of possible partners. All analyses were carried out in the R package *bipartite* (Dormann et al., 2008), using the functions *plotweb* and *specieslevel*. Following the approach of Gómez et al. (2015), we used the quantitative modularity (*Q*) of the bipartite networks to explore the occurrence of different photobiont niches in the species of the *R. decipiens* group. Optimal modular configuration was calculated using the Beckett algorithm (Beckett, 2016) as implemented in the *computeModules* function of the *bipartite* package. The algorithm was run 20 times with 10^8^ MCMC steps and a tolerance level of 10^–10,^ retaining the iteration with the highest likelihood value as the optimal modular configuration. In order to check for significance, we built a null model based on 500 random networks calculated using the function *nullmodel* and the *vaznull* null model which implements the null model proposed by Vázquez et al. (2007) in which matrix connectance remains the same as in the original network but total marginals change. We calculated modularity for each of the random matrices and calculated a *z*-score as (X*_observed_* − μ*_null_*)/σ_null_, being X*_observed_* the actual value of the parameter, μ*_null_* the mean of the parameter for the population of matrices in the null model, and σ_null_ their standard deviation. *p*-values for the *z*-score were calculated as the number of elements of the null model showing higher or lower values than the observed value divided by the total number of elements in the null model. To explore if the resulting modules were a consequence of the species geographic distribution we compared the geographic distances within and between modules. This was done with PERMANOVA tests using the *adonis* function of the *vegan* R package (Oksanen et al., 2012). The geographic distance matrix was obtained with the *distm* function of the *geosphere* R package (Hijmans et al., 2017).

### Genetic Diversity Statistics

We calculated genetic diversity statistics at both the island and *Ramalina* species levels. We used the software DnaSP v.6.12.01 to calculate two indices: haplotype diversity (*Hd*, Nei, 1987), and nucleotide diversity (*π*, Nei, 1987). *π* was calculated using the Jukes and Cantor correction (Lynch and Crease, 1990). Instead of using the raw ASVs abundance table as input, we generated DNA alignments for each species and each island, which included a representative sequence of each ASV present in each sample. The two indices were then calculated for each alignment.

### Phylogenetic Signal

We used the phylogeny of the *R. decipiens* group which will be published in a forthcoming paper (Blázquez et al. unpublished) to calculate the phylogenetic signal of the specialization, species degree, ND, haplotype diversity and nucleotide diversity. We employed two different methods to calculate the phylogenetic signal: Pagel’s *λ* (Pagel, 1999) as implemented in the *phylosig* function of the *phytools* R package (Revell, 2012) and Blomberg’s *K* (Blomberg et al., 2003) as implemented in the *phylosignal* function of the *picante* R package (Kembel et al., 2010). Pagel’s λ can have values between 0 (no phylogenetic signal) and 1 (Brownian motion model, that is, correlation among trait values is proportional to the extent of shared ancestry). Blomberg’s *K* can be interpreted as follows: if *K* = 1 the trait under analysis follows a mode of evolution that is consistent with Brownian motion. If K > 1 close relatives are more similar than expected under Brownian motion. On the other hand, in K values <1 close relatives are less similar than expected (Blomberg et al., 2003).

## Results

### Phylogenetic Relationships, Haplotype Networks, and Frequency of ASVs

**Figure 1:**
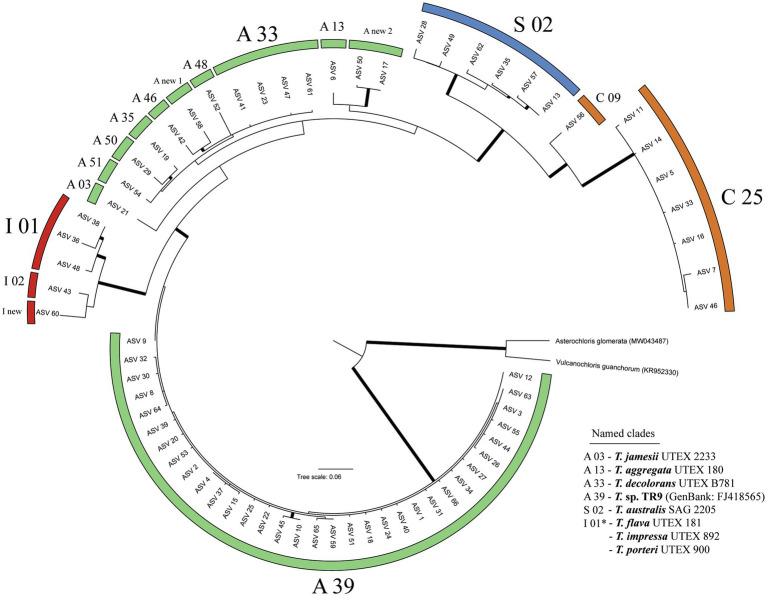
RAxML phylogeny of the 66 ASVs. Branches with bootstrap support higher than 70 are highlighted in bold. Clade nomenclature follows Muggia et al. (2020). Formally described *Trebouxia* lineages are indicated with UTEX or SAG culture numbers.

**Figure 2:**
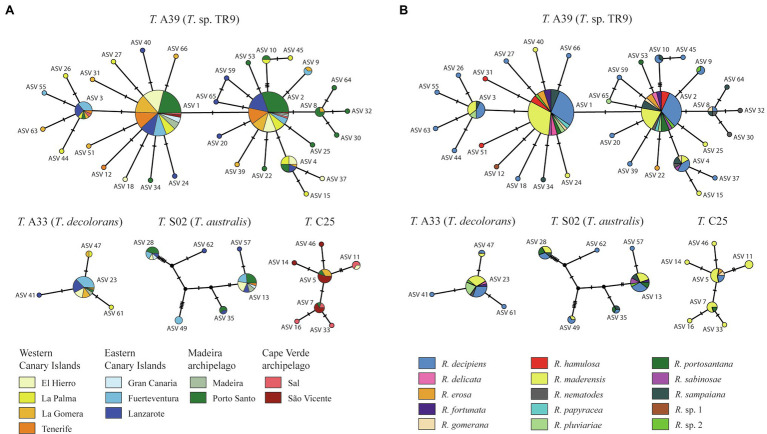
TCS networks of the ASVs colored according to **(A)** island and **(B)** mycobiont species. The size of the circles is proportional to the number of samples carrying the ASV. Black-filled circles indicate missing haplotypes.

We obtained a total of 2,949,259 raw reads, of which 2,175,408 passed DADA2 quality filter. After removing ASVs represented by less than 100 reads, 66 ASVs remained (Figure 1). The 66 ASVs clustered into the four main *Trebouxia* clades (Leavitt et al., 2015; Muggia et al., 2020), but not into the newly reported clade D (Xu et al., 2020). 62 ASVs belonged to four formally described species (i.e., *Trebouxia jamesii*, *T. aggregata*, *T. decolorans,* and *T. australis*) and to nine of the undescribed *Trebouxia* lineages reported by Muggia et al. (2020). Four ASVs did not match with any of the sequences assembled by Muggia et al. (2020). These ASVs represented three new lineages, two of them in clade A and one in clade I. Thirty-three of the 66 ASVs clustered into *Trebouxia* A39. This clade was identified as *Trebouxia* sp. TR9 through its reference sequence (FJ418565). The most common photobionts found in this study were ASVs 1 and 2, both belonging to *Trebouxia* sp. TR9 (Figure 2) and diverging by just one nucleotide. They were present on all islands and often coexist in the same localities. These two ASVs were the most common photobionts in the Canarian and Madeira archipelagos, together they represented the main photobionts in 78 and 85% of the Canarian and Madeiran thalli, respectively. Regarding the Cape Verde archipelago, the main photobionts from all samples corresponded to one of the five ASVs placed in the *Trebouxia* clade C25. On the other hand, 41 ASVs were only found in one thallus, although only 6 of them acted as the main photobiont.

ASVs belonging to *Trebouxia* clades A and C were found in all archipelagos. Clades I and S were not found in Cape Verde samples. Mycobiont identity had a limited effect on the frequency of ASVs (Table 1). Of the 25 ASVs present in more than one thallus only one, belonging to the species *Trebouxia jamesii*, was significantly associated to some *Ramalina* species. The island of origin had a greater influence on ASV frequency, with a significant effect in 8 of the 25 included ASVs. The greatest effect was observed in ASVs 5, 7, and 11. These three ASVs belong to *Trebouxia* clade C 25 and act as main photobionts in thalli collected in Cape Verde, but also occur in small numbers in El Hierro, La Gomera, and Porto Santo. An interaction between island an mycobiont identity was found in ASV 9. However, this ASV only occurs in three thalli: Two specimens of *R. decipiens* collected in Fuerteventura and one of *R*. sp. 2 collected in La Gomera.

**Table 1:** *p-*values of two-way ANOVA and Kruskal-Wallis test exploring the effect of island and/or mycobiont species on the frequency of each ASV.

ASV	*P* species	*P* island	*P* species*island
ASV 1	0.8864	**0.0028** **	0.4886
ASV 2	0.2556	0.4255	0.2016
ASV 3	0.5121	0.0803	0.7751
ASV 4	0.0831	0.9234	0.9996
ASV 5	0.5794	**4.73E-14** ***	-
ASV 6	0.9861	0.1584	0.3857
ASV 7	0.6763	**6.17E-12** ***	-
ASV 8	0.5544	0.8430	0.6702
ASV 9	0.1188	**0.0007** ***	**2.85E-12** ***
ASV 10	0.9892	0.4981	0.6184
ASV 11	0.9162	**6.26E-08** ***	-
ASV 13	0.9886	0.9360	0.9964
ASV 19	0.5398	0.0879	0.9068
ASV 21	**1.25E-03** ***	**0.0011** **	-
ASV 23	0.8087	0.1121	0.9435
ASV 28	0.1180	0.9886	0.9997
ASV 29	0.9410	0.1643	0.5964
ASV 35	0.9788	0.9284	0.9924
ASV 36	**0.0125** *	0.6126	0.3036
ASV 38	0.7826	0.2845	0.2359
ASV 42	**1.12E-02** ***	0.8119	-
ASV 43	0.0909	**0.0006** ***	-
ASV 47	1.0000	0.4689	0.9905
ASV 49	0.9996	**0.0105** **	0.9507
ASV 54	0.9831	0.2656	0.5889

ASVs lacking the island-species interaction are those who did not meet the assumptions necessary to perform a two-way ANOVA. Bold values are statistically significant.

* < 0.05;

** < 0.01;

*** <0.001.

### Photobiont Community Structure

**Figure 3:**
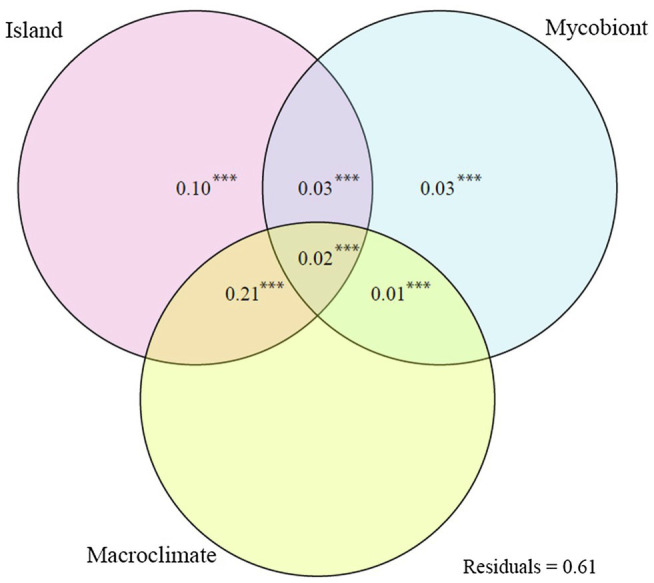
Venn diagram showing variance partitioning between the simple and combined effects of the explanatory variables of the dbRDA (island, mycobiont identity, and macroclimate) and their mixed effects. The explained variation indicated are the adjusted R^2^ values. Values <0 not shown. ***p < 0.001.

The dbRDA analysis returned a *F* = 3.265 with a *p* = 0.001. The constrained axes explained 0.34 of the variance in ASV community structure (Figure 3). Island was the variable with the greatest simple effect. On the other hand, macroclimate was the variable with the smallest simple effect. However, the main driver of ASV community structure was the mixed effect of island and macroclimate, explaining a greater variance proportion than all other simple and mixed effects combined. Mycobiont identity was the least important variable, taking its simple and mixed effects into account.

### Co-occurrence Analyses and Frequency of ASVs

Out of 197 thalli studied, 102 showed coexistence of several ASVs (51.77%). Specimens from the Canary Islands and Cape Verde showed a higher proportion of multiple ASVs within the thallus (56 and 58%, respectively) than specimens from the Madeira archipelago (38%). Algal coexistence was observed in all species (Table 2) except *Ramalina* sp. 1, which is most likely an artifact given that the species is represented in our dataset by only one specimen. The mean number of ASVs per thallus was 2.19, with a median of 2. Mean ASV count per thallus ranged from 1.30 in *Ramalina hamulosa* to 4.67 in *Ramalina pluviariae*. The sample with the highest ASV count was a *Ramalina maderensis* collected in Lanzarote (SPO 6337) containing 10 algae. However, on average the main photobiont amounted to 94% of the reads in thalli showing algal coexistence. The mean abundance of the main photobiont in thalli showing coexistence ranged from 83% in *Ramalina erosa* to 99% in *Ramalina* sp. 2. There were only six thalli, of varied origin and representing four *Ramalina* species, in which the main photobiont accounted for less than 70% of the reads. In most of them, the main and secondary photobionts were different ASVs belonging to the same *Trebouxia* species.

**Table 2:** Summary of algal coexistence across *Ramalina* species.

Species	N° thalli	% thalli >1 ASV	Mean ASV count/thallus	Maximum n° ASV/thallus	Minimum % main photobiont	Mean % main photobiont	Max % main photobiont
*Ramalina decipiens*	57	60%	2.46	9	42.93%	93.85%	99.94%
*Ramalina delicata*	3	33%	2.33	5	95.89%	95.89%	95.89%
*Ramalina erosa*	9	22%	1.22	2	67.04%	83.50%	99.96%
*Ramalina fortunata*	8	38%	1.38	2	99.59%	99.81%	99.95%
*Ramalina gomerana*	3	33%	3	7	85.78%	85.78%	85.78%
*Ramalina hamulosa*	10	30%	1.3	2	95.84%	97.99%	99.81%
*Ramalina maderensis*	57	53%	2.23	10	54.28%	93.74%	99.98%
*Ramalina nematodes*	6	50%	2	5	90.28%	96.63%	99.87%
*Ramalina papyracea*	6	50%	2	4	96.19%	98.33%	99.63%
*Ramalina pluviariae*	6	100%	4.67	7	83.74%	93.73%	99.79%
*Ramalina portosantana*	8	50%	2	5	88.48%	94.70%	99.95%
*Ramalina sabinosae*	4	100%	2.25	3	80.00%	94.22%	99.75%
*Ramalina sampaiana*	13	38%	1.92	6	69.96%	90.89%	98.60%
*Ramalina* sp. 1	1	0%	1	1	-	-	-
*Ramalina* sp. 2	6	50%	1.5	2	99.85%	99.89%	99.96%

Number of studied thalli, percentage of thalli that showed algal coexistence, minimum, mean, and maximum number of ASVs per thallus and minimum, mean, and maximum relative abundance of the main photobiont across thalli showing algal coexistence.

### Bipartite Network, Specialization, and Modularity

**Figure 4:**
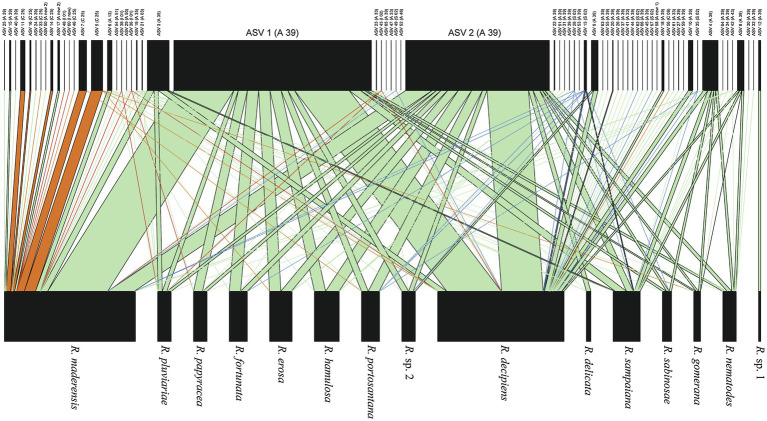
Bipartite network based on rarefied reads showing interactions between Ramalina species and *Trebouxia* ASVs. Interactions with ASVs belonging to *Trebouxia* clades A, C, I, and S are depicted in green, orange, red and blue, respectively. Width of the links is proportional to the frequency of the association.

**Figure 5:**
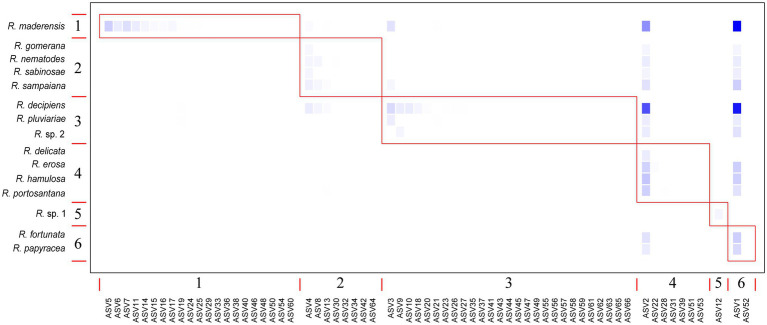
Modularity plot featuring the six modules identified by the Becket algorithm (*Q* = 0.179, z-score = 709.31; value of *p* <0.001) in the rarefied reads network. Taxa are sorted according to their modular affinity, mycobiont species as rows, and photobiont ASVs as columns. Red boxes delineate the six modules. Color intensity is proportional to the relative abundance of each ASV per mycobiont species.

The bipartite interaction networks (Figure 4; Supplementary Figure 1) clearly depict ASVs 1 and 2 as the main photobionts of the *Ramalina decipiens* group. *Ramalina* sp. 1 is the only species not associated with these ASVs. This is most probably due to the poor representation of this species in the dataset. Excluding *Ramalina* sp. 1, whose only thallus associates with another ASV belonging to *Trebouxia* sp. TR9, specialization (*d’*) based on rarefied reads varied from 0.98 in *Ramalina delicata* to 0.51 in *Ramalina maderensis*. Species degree varied from 37 in *Ramalina decipiens* to 4 in *Ramalina erosa* and *ND* varied from 0.56 in *Ramalina decipiens* to 0.06 in *Ramalina erosa*. Species degree and ND were highest in the two most sampled species (*R. decipiens* and *R. maderensis*). Specialization parameters obtained from the data non considering read counts returned similar results for species degree and ND, but overall lower values of *d’*, that ranged from 0.59 in *R. erosa* to 0.27 in *R. decipiens*. All specialization parameters are summarized in Table 3. The network based on rarefied reads was significantly modular (*Q* = 0.18, z-score = 24.75, value of *p* <0.001), and detected the presence of six modules (Figure 5). The modularity analyses of the second network resulted in a non-modular pattern (*Q* = 0.21, z-score = −0.68, value of *p* = 0.241). The modular structure recovered in the first analysis seems to be a consequence of the mycobiont species distributions inasmuch as the geographic distances between and within modules were significantly different (*F* = 7.98, *R^2^* = 0.17, *p* < 0.001).

**Table 3:** Summary of specialization parameters of the bipartite networks based on the sum of the rarefied reads of the thalli of each species and the conversion of the abundance of ITS2 reads of the thalli to presence-absence data.

	Rarefied reads network			Presence-absence network		
Mycobiont species	*d’*	Species degree	*ND*	*d’*	Species degree	*ND*
*Ramalina decipiens*	0.54	37	0.56	0.28	38	0.58
*Ramalina delicata*	0.98	5	0.08	0.46	5	0.08
*Ramalina erosa*	0.68	4	0.06	0.59	4	0.06
*Ramalina fortunata*	0.72	5	0.08	0.54	5	0.08
*Ramalina gomerana*	0.56	7	0.11	0.38	7	0.11
*Ramalina hamulosa*	0.70	6	0.09	0.53	6	0.09
*Ramalina maderensis*	0.51	33	0.50	0.29	33	0.50
*Ramalina nematodes*	0.51	9	0.14	0.33	9	0.14
*Ramalina papyracea*	0.74	6	0.09	0.46	6	0.09
*Ramalina pluviariae*	0.54	12	0.18	0.31	12	0.18
*Ramalina portosantana*	0.70	8	0.12	0.41	8	0.12
*Ramalina sabinosae*	0.58	5	0.08	0.42	6	0.09
*Ramalina sampaiana*	0.51	14	0.21	0.33	14	0.21
*Ramalina* sp. 1	0.62	6	0.09	0.44	6	0.09
*Ramalina* sp. 2	1.00	1	0.02	1.00	1	0.02

### Genetic Diversity Statistics

**Figure 6:**
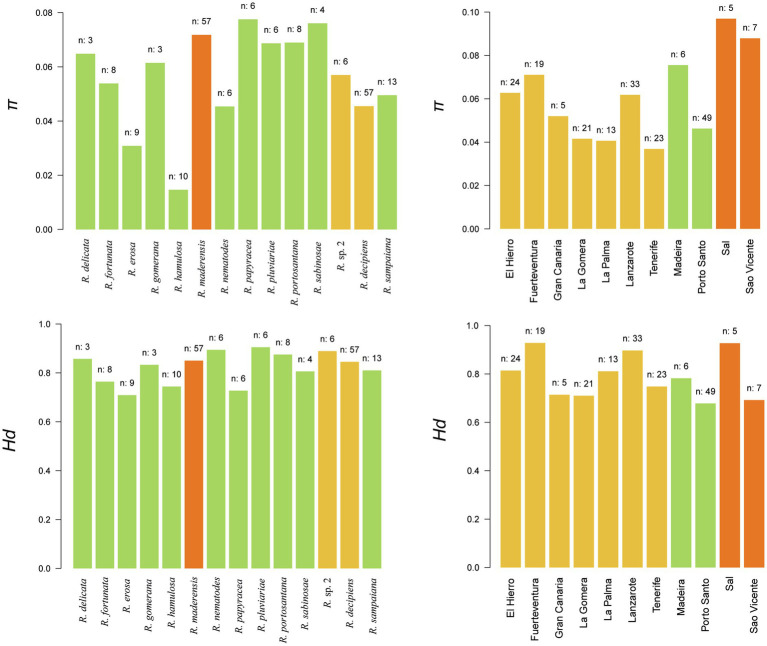
Diversity statistics. Upper and lower panels represent nucleotide (*π*) and haplotype (*Hd*) diversities at the species (left) and island (right) levels. Species plots are colored by the number of archipelagos in which the species is present: green (one), yellow (two), and orange (three). Island plots are colored by archipelago: yellow (Canarian archipelago), green (Madeira archipelago), and orange (Cape Verde archipelago). The number of samples by which the island or species is represented in the dataset appears on top of each bar.

Nucleotide diversity (*π*) and haplotype diversity (*Hd*) were calculated at the island and species levels (Figure 6). At the island level, *π* ranged from 0.036 in Tenerife to 0.096 in Sal. *Hd* values ranged from 0.678 in Porto Santo to 0.928 in Fuerteventura. On average, Cape Verde had the highest *π* value (0.092) followed by Madeira (0.060) and the Canary Islands (0.052). Regarding *Hd*, Cape Verde again showed the highest value (0.810), followed by the Canary Islands (0.803) and Madeira (0.730). At the species level, *π* values ranged from 0.014 in *Ramalina hamulosa* to 0.077 in *Ramalina papyracea*. *Hd* values ranged from 0.709 in *Ramalina erosa* to 0.905 in *Ramalina pluviariae*.

### Phylogenetic Signal

The parameters *d’*, species degree, and *ND* derived from both networks were not phylogenetically structured according to Pagel’s *λ* and Blomberg’s *K*. Regarding genetic diversity, neither *π* nor *Hd* were phylogenetically structured either. Phylogenetic signal statistics are summarized in Table 4.

**Table 4:** Summary of phylogenetic signal statistics.

	*λ*	value of *p* (*λ*)	*K*	value of *p* (*K*)
*d’* (rarefied reads network)	0.716	0.535	0.771	0.183
Species degree (rarefied reads network)	0.257	0.634	0.734	0.227
*ND* (rarefied reads network)	0.257	0.634	0.734	0.218
*d’* (presence-absence network)	0.000	1.000	0.596	0.163
Species degree (presence-absence network)	0.237	0.665	0.735	0.209
*ND* (presence-absence network)	0.237	0.665	0.735	0.227
*π*	0	1	0.414	0.631
*Hd*	0	1	0.423	0.616

## Discussion

We explored the role of the photosynthetic partners in the radiation of a clade of symbiotic organisms. According to our knowledge, this is the first time that the trophic niche is analyzed within the evolutionary framework of an island radiation in lichen-forming fungi. We found no differences in the identity of the photobionts associated with the species of the *R. decipiens* group. The vast majority of specimens analyzed contained as their main photobiont one of the two most abundant ASVs, both belonging to *Trebouxia* sp. TR9. The frequency of both ASVs does not differ among *Ramalina* species, and we have indistinctly found both acting as major photobionts in thalli collected from the same locality on multiple occasions. The prevalence of *T.* sp. TR9 as main photobiont is not exclusive of the *R. decipiens* group. It was found in Macaronesian specimens of *R. farinacea* using Sanger sequencing (del Campo et al., 2013) and NGS (Molins et al., 2021). BLAST searches of the two predominant ASVs revealed their presence also in other genera. Both appear in *Protoparmelia montagnei* thalli in southern Spain (Singh et al., 2017) while ASV2 additionally appears in *Lecanographa amylacea* thalli in Poland and Sweden (Ertz et al., 2018). The fact that *T.* sp. TR9 is the main photobiont of *Ramalina* species in Macaronesia is congruent with our current knowledge of its physiology. Casano et al. (2011) showed that *T*. sp. TR9 grew faster and had better photosynthesis performance in *in vitro* experiments under relatively high temperatures and irradiances than *T. jamesii*, the main photobiont of *R. farinacea* thalli in the Iberian Peninsula (del Campo et al., 2013; Molins et al., 2021). The presence of both ASVs in southern Spain (Singh et al., 2017) also seems to point to the fact that this species can cope with higher temperature and light intensity given the similarity of that locality to those in which the *R. decipiens* group species occur in Macaronesia. Surprisingly, the records from Poland and Sweden occurred in localities with radically different climatic conditions (Ertz et al., 2018). Lichen photobionts can show different physiologies even when they are phylogenetically very close (Sadowsky et al., 2016). The presence of ASV2, but not ASV1, in climatically divergent localities, such as the Białowieża Primeval forest in Poland or the island of Gotland in Sweden (Ertz et al., 2018), might suggest that they have differences in ecophysiological adaptations. Their presence in thalli collected in a same locality could be reflecting climatic differences at the microsite scale, as we have detected differences up to 4°C in two rocky outcrops separated by only 200 m (data not shown). The existence of such physiological differences will be explored in a forthcoming study.

We did not find differences in photobiont dominance between mycobiont species either. We detected algal co-occurrence in c. 50% of the studied thalli, a phenomenon reported in different lineages of lichen-forming fungi (Blaha et al., 2006; Piercey-Normore, 2006; del Campo et al., 2010; Muggia et al., 2014; Rolshausen et al., 2018). Casano et al. (2011) showed that the two *Trebouxia* species detected in *R. farinacea* apparently displayed complementary physiological behaviors, arguing that algal co-occurrence in this species could have allowed it to expand its geographical range. However, most photobiont co-occurrence reports have been based on Sanger sequencing, so the relative abundance of each photobiont could not be assessed. Recently, Molins et al. (2021) revisited photobiont diversity in *R. farinacea* with NGS data showing that in c. 70% of all thalli one *Trebouxia* species was dominant. Paul et al. (2018) reported similar results for *Umbilicaria hispanica* and *U. pustulata*, with the main photobiont being represented by at least 80% of the reads in c. 80% of all thalli. In our study, this proportion is even more pronounced, with the main photobiont being dominant in 94% of the thalli showing algal co-occurrence. The overwhelming dominance of the main photobiont over the rest sheds doubt on the ecological importance of algal co-occurrence. Photobiont co-occurrence has been studied more in-depth in coral-dinoflagellate symbioses. Young corals establish symbiosis with a subset of the photobionts available in their environment (Poland et al., 2013; Yamashita et al., 2013). This first photobiont community is restructured in response to environmental heterogeneity (Rowan, 2004) or other processes, such as re-establishment of photobiont communities after a previous stress-induced loss (Jones et al., 2008). After the restructuration process, adult corals usually end up with a single Symbiodiniaceae lineage dominating the photobiont community (Parkinson and Baums, 2014). This agrees with the results shown by Molins et al. (2021) for *Ramalina farinacea* in which young and old thalli showed different microalgal communities, more diverse in young thalli.

The community structure of the photobionts associated to species of the *R. decipiens* group is chiefly explained by the interaction between island and macroclimate, mycobiont identity being only of marginal importance. This finding agrees with previous studies that have reported macroclimate as a major driver of genetic variation of photobionts (Beiggi and Piercey-Normore, 2007; Nelsen and Gargas, 2009; Fernández-Mendoza et al., 2011; Leavitt et al., 2016; Magain et al., 2017). Other studies, however, have suggested a greater importance of mycobiont identity (Buckley et al., 2014; Leavitt et al., 2015; Vančurová et al., 2018; Jüriado et al., 2019). The relative importance of both factors can be influenced by the scale of the study. For instance, Pino-Bodas and Stenroos (2021) studied the diversity of the photobionts associated with the genus *Cladonia* by Sanger sequencing of the ITS and actin type I genes and found that the relative importance of the different factors differed between the two genes. However, when the ITS dataset was trimmed to include only the specimens for which actin type I sequences were available (losing mycobiont species in the process) the results for both datasets were similar (Pino-Bodas and Stenroos, 2021). The importance of the island-macroclimate interaction in our study is congruent with the climatic heterogeneity of Macaronesia, as the different islands show marked differences in the vegetation assemblages they harbor (del Arco Aguilar and Rodríguez Delgado, 2018) but at the same time, due to their pronounced orography, close areas can experience radically different climatic conditions (Sánchez-Benítez et al., 2017).

We found no phylogenetic signal in either the specialization parameter *d’* or the genetic diversity indices tested, suggesting that species specialization toward their photobionts is not an evolutionarily conserved trait. Magain et al. (2017) studied patterns of mycobiont specialization toward their cyanobionts in the *Peltigera* section *Polydactylon*. They found that generalist and specialist species were distributed all over the phylogeny proposing that photobiont specialization by lichen-forming fungi does not equal to an evolutionary dead-end as it has been proposed for other organisms (e.g., Haldane, 1951; Nosil and Mooers, 2005). Similar results of no phylogenetic conservatism in mycobiont specialization toward photobionts have been recently reported by Pino-Bodas and Stenroos (2021) for the genus *Cladonia.* Our results suggest that the *R. decipiens* group species associate with locally adapted photobionts, as do other lichen-forming fungi (Blaha et al., 2006; Muggia et al., 2014; Rolshausen et al., 2018). This is particularly clear in *R. maderensis*, the only species of the group occurring in the Cape Verde archipelago. In Cape Verde, *R. maderensis* has performed a photobiont switch and associates predominantly with ASVs belonging to *Trebouxia* C25. *Trebouxia* C25 has only been found previously in *Parmotrema aldabrense* thalli in Kenya, in a locality near the coast (Muggia et al., 2020). Nothing is known about this species physiology but the fact that it occurs mainly in tropical regions as well as all other clade C *Trebouxia* species in Muggia et al. (2020), could be indicative of a physiology adapted to higher temperatures and light intensities than those in which *T.* sp. TR9 thrives.

We have not observed differences neither in identity nor in the abundance of ITS2 reads in the photobionts associating with the different species of the *R. decipiens* group. Because differences in identity can be linked with differentiated growth rates and photosynthetic performances at the species (Casano et al., 2011) and strain (Determeyer-Wiedmann et al., 2019; Aigner et al., 2020) levels, we conclude that a partitioning of the trophic niche has probably not occurred during the *R. decipiens* group radiation. This fact, coupled with the marginal importance of mycobiont species on photobiont community structure and the geographic structure underlying the bipartite network modules, suggests that the photobionts are not a factor driving the *R. decipiens* group diversification. A segregation of the trophic niche is a key driver of adaptive radiation in many other examples, such as the Darwin finches (Grant and Grant, 2008), East African cichlid fishes (Danley and Kocher, 2001), *Tylomelania* freshwater gastropods (Rintelen et al., 2004), *Cyprinodon* pupfishes (Martin and Feinstein, 2014), *Tetragnatha* spiders (Kennedy et al., 2019), and many others (Gillespie et al., 2020). However trophic niche differentiation is not the only factor that can explain adaptive radiation. It has been hypothesized that Hawaiian lobeliads have diversified physiologically across environments differing in a key resource (i.e., light, Montgomery and Givnish, 2008). Recently it has been proposed (Gillespie et al., 2020) that symbiotic interactions with microbes could facilitate adaptive radiation. Physiological speciation in the *R. decipiens* group and a study of the species associated microbiomes will be explored in forthcoming works.

## Conclusion

We have found no evidence for trophic niche segregation in the context of the diversification of the Macaronesian endemic species of the *R. decipiens* group. They do not differ neither in the identity nor in the abundance of their photobionts. Thus, we argue that the photobionts are not a key factor driving speciation in the group. Instead, *Ramalina* species appear to associate with locally adapted *Trebouxia*. Algal coexistence was common, but the contribution of the secondary photobionts was marginal in most thalli. This opens new questions about the ecological importance of algal coexistence.

## Data Availability Statement

The datasets presented in this study can be found in online repositories. The names of the repository/repositories and accession number(s) can be found in the article/**Supplementary Material**.

## Author Contributions

SP-O, FG, IP-V, and MB designed the study. MB, IP-V, and SP-O organized the fieldwork, research permits, and collected the specimens. MB and LH-M performed the laboratory work. MB analyzed the data and wrote the manuscript. All authors contributed to the article and approved the submitted version.

## Funding

This study was financed by grant CGL2016-81136-P from the Spanish Ministry of Science and Innovation. MB was supported by grant BES-2017-081807. SP-O was supported by the grant RYC-2014-16 784 from the Spanish Ministry of Economy, Industry and Competitiveness.

## Conflict of Interest

The authors declare that the research was conducted in the absence of any commercial or financial relationships that could be construed as a potential conflict of interest.

## Publisher’s Note

All claims expressed in this article are solely those of the authors and do not necessarily represent those of their affiliated organizations, or those of the publisher, the editors and the reviewers. Any product that may be evaluated in this article, or claim that may be made by its manufacturer, is not guaranteed or endorsed by the publisher.

## Footnotes

^1^ http://tree.bio.ed.ac.uk/software/figtree/

## Supplementary Material

SUPPLEMENTARY FIGURE 1 Bipartite network based on presence-absence data showing interactions between Ramalina species and *Trebouxia* ASVs. Interactions with ASVs belonging to *Trebouxia* clades A, C, I, and S are depicted in green, orange, red and blue, respectively. Width of the links is proportional to the frequency of the association.
