# Supplementary figures and images for: The Role of Photobionts as Drivers of Diversification in an Island Radiation of Lichen-Forming Fungi
Source: Front Microbiol. 2022 Jan 3;12:784182. doi: 10.3389/fmicb.2021.784182 (PMC8763358; doi:10.3389/fmicb.2021.784182)

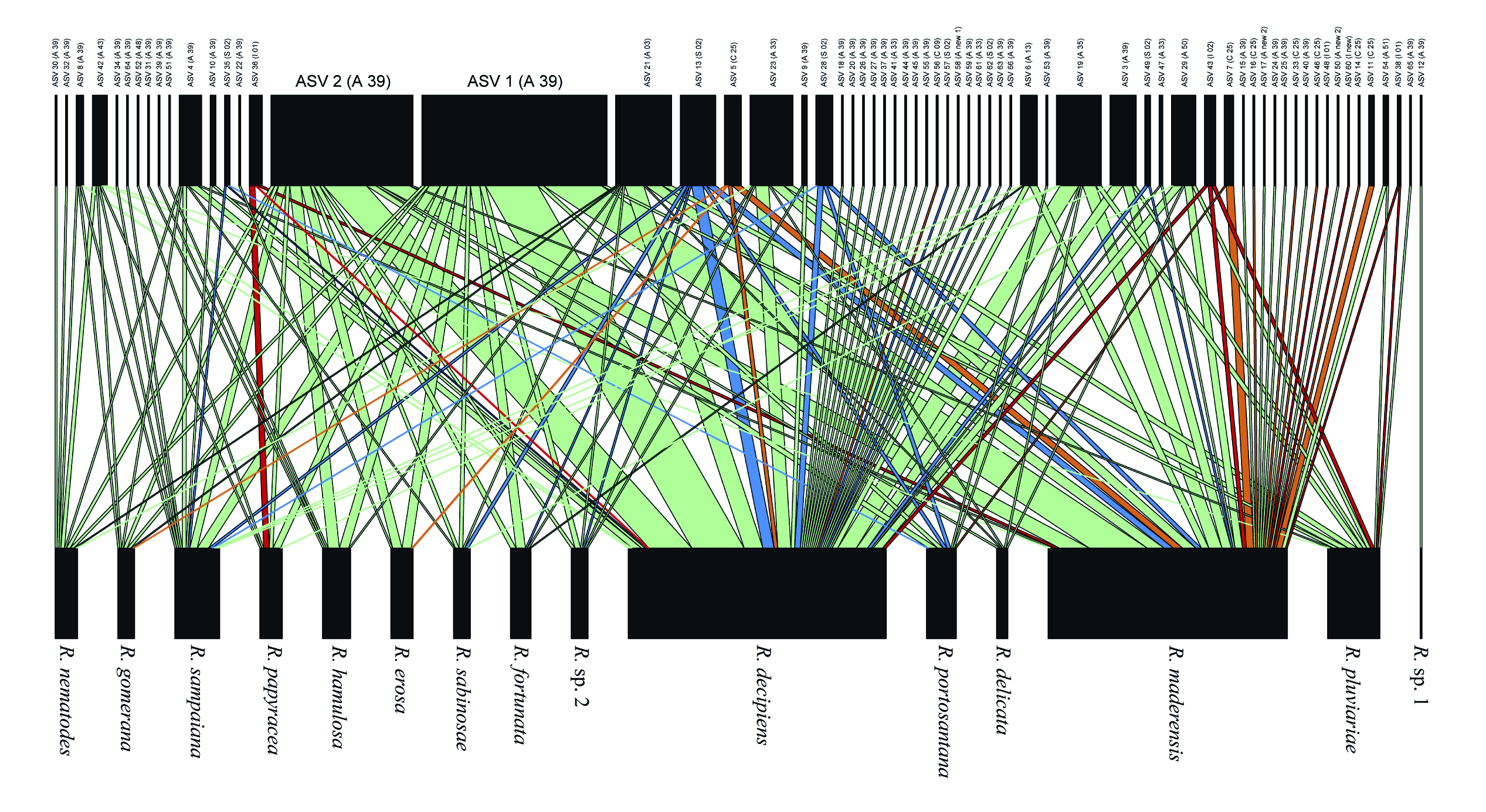

Supplement: SUPPLEMENTARY FIGURE 1 — Bipartite network based on presence-absence data showing interactions between Ramalina species and Trebouxia ASVs. Interactions with ASVs belonging to Trebouxia clades A, C, I, and S are depicted in green, orange, red and blue, respectively. Width of the links is proportional to the frequency of the association. [file Image_1.tif]
